# The Development and Implementation of New Assessment Tools for the Surgical Clerkship Rotation

**DOI:** 10.15694/mep.2018.000004.1

**Published:** 2018-01-04

**Authors:** Mila Kolar, Eleni Katsoulas, Ayca Toprak, Theresa Nowlan-Suart, Lindsay Davidson, Andrea Winthrop, Amber Hastings-Truelove, Denise Stockley

**Affiliations:** 1 Queen's University

**Keywords:** Assessment tool, Rubric, Clerkship

## Abstract

This article was migrated. The article was marked as recommended.

**Innovation:** We developed two new rubrics with explicit behavioural anchors to assess students in the Queen’s undergraduate medical education (UGME) surgery clerkship rotation. These rotation rubrics, complemented by a new ambulatory clinic encounter card, improved the quality, consistency, and timeliness of feedback for clerks from faculty preceptors. This innovation was introduced during a comprehensive workplace-based assessment re-design being undertaken in the Department of Surgery as part of the transition to a post-graduate competency-based medical education (CBME) system for post-graduate education (PGME). The core UGME working group, comprised of a faculty surgeon, assessment consultant, and a surgical resident, selected terminology and designed the tool visual structure to be similar to the new post-graduate assessment tools, since most preceptors supervise learners in both programs. This consistency enhanced buy-in from faculty and ensured a smooth transition to the use of the new UGME tools.

**Development:** The new assessment process was developed and piloted in three phases: (1) development of an assessment system based on rubrics with explicit behavioural descriptors as the key assessment tools; (2) implementation of a pilot study to establish the acceptability and feasibility of the use of these rubrics, with iterative revisions based on stakeholder feedback; and (3) development of a validity argument for the use of these assessment tools. The latter is scheduled for 2018.

**Outcomes:** The use of these rotation behaviour-anchored rubrics and corresponding ambulatory clinic encounter card has greatly improved the mid- and final-rotation feedback provided to students on the Surgery Clerkship. The concrete, descriptive information provided by the rubrics allows the course director to provide specific feedback during rotation exit meetings. The course director has the ability to clearly articulate to students the areas where they have met (or exceeded) the expected level of competency, as well as areas which require additional attention.

## Introduction

Research into improving assessment methods in medical education and related health sciences fields has become particularly relevant with the national and international movement towards Competency-based Medical Education (CBME) ( Bould, Crabtree, & Naik, 2009; Driessen, van Tartwijk, Govaerts, Teunissen, & van der Vleuten, 2012; Harris, Snell, Talbot, & Harden, 2010; Quinn et al., 2015). Assessment is one of the key elements that facilitates this transition ( Scheele et al., 2008; Holmboe, Sherbino, Long, Swing, & Frank, 2010; Driessen& Scheele, 2013). CBME has changed the way medical educators think about assessment ( Carraccio & Englander, 2013; Holmboe et al., 2010). At Queen’s University School of Medicine, as part of our undergraduate medical education (UGME) competency-based curricular framework, we have focused on improving formative assessment tools to provide more explicit feedback about student performance. This approach provides students with constructive feedback to support their learning. Well-constructed formative workplace-based assessment tools also meet the increasing demand to accurately document direct observation of learners’ performance in the clinical learning environment.

Reframing medical education within the CBME context, with the use of Entrustable Professional Activities (EPAs) requires changes to current systems of assessment, including increased frequency of formative and summative assessments. Many of the current types of workplace-based assessment utilized during clerkship rely on checklists or Likert-type scales ( Sharma, Cui, Leighton, & White, 2012; Lee & Wimmers, 2011; Haffling, Beckman, & Edgren, 2011). While checklists are simple and easy to use, feedback is limited to what was or was not done by the learner. As Toprak et al point out ( Toprak, Luhanga, Jones, Winthrop, and McEwen 2016), checklists do not measure the quality of learners’ performances and they often lack the type of feedback explicitly explaining how learners could improve in the future. Likert-type scales provide slightly more information than done/not done, but the performance standard for each point on the scale is implicit; where any student falls on a 5- or 7-point scale may be interpreted differently by different assessors ( Gingerich, Regehr, & Eva, 2011; Kogan, Conforti, Iobst, & Holmboe, 2014). McManus, Thompson, and Mollon (2006) found as much as a 12% difference in the leniency and stringency of assessors. The potential for assessor variability makes it difficult for course directors or undergraduate preceptors to make accurate judgments about a learner’s progress when reviewing forms from multiple assessors ( Driessen& Scheele, 2013). One way to overcome the leniency/stringency effect is to utilize a rubric-based assessment tool ( Driessen& Scheele 2013).

Queen’s UGME has been increasing the use of rubrics throughout its Clerkship curriculum over the last several years, as assessment tools have been revised. Rubrics are assessment instruments that explicitly define criteria of performance in terms of increasing complexity using behavioural descriptors (behavioural anchors) ( Jönsson, Svingby, & Attström, 2007). While there is still a Likert-type scale, the explicit behavioural anchors guide assessors on the use of the Likert scale but providing a frame of reference. Advantages of rubrics include: (a) shared frames of reference for assessors through the use of a clear assessment framework and standards of performance guide; (b) improved consistency, reliability and efficiency of scoring for both single and multiple assessors; (c) improved reliability and validity in scoring complex performance; (d) immediate feedback on trainee performance; and (e) improvement in trainees’ ability to self-assess performance ( Jönsson, et al., 2007; Reddy & Andrade, 2010). According to Jönsson et al. (2014), rubrics can improve students’ learning and accuracy of self-assessment, evaluator’s accuracy and efficiency in assessment, and ultimately result in more successful students.

This report summarizes our assessment re-design in the Surgical Clerkship. The goal of this effort was to improve the system of workplace-based assessment in the Queen’s UGME Surgery Clerkship rotation. In addition to providing students with more concrete, actionable feedback, the change was motivated by the planned adoption of Competency-based Medical Education and an Entrustable Professional Activities (EPA) framework from the Association of Faculties of Medicine of Canada which was adopted by all Canadian medical schools in 2016.

The pilot of our new assessment tools was initiated in July 2015 with the development of a new assessment system for the Surgery Clerkship Rotation based on rubrics with explicit behavioural descriptors. A working group made up of the Surgery Clerkship Course Director, the UGME assessment consultant, the UG Teaching, Learning and Integration Director, the Clerkship Director, and a surgical resident developed two new behavioural-anchored rubrics to be used during the Surgical Clerkship. This process took in to account, and was consistent with what was being implemented in Queen’s post-graduate programs transitioning to a competency-based medical education (CBME) model. Development of the new rotation rubric was followed by a pilot implementation, which included the development of an additional ambulatory clinic encounter card. The last phase of our pilot will be the development of a validity argument for use of these tools, and this is not included in this current report.

## Development

We developed our new clinical rotation rubrics as part of a workplace-based system of assessment to assist faculty in identifying students’ patterns of performance over time, and to facilitate documentation of this progress. The working group developed the tools based on the Queen’s School of Medicine Curricular Goals and Competency-Based Objectives (2014). These objectives are informed by the Pan-Canadian Entrustable Professional Activities (2016), the Clinical Presentations of the Medical Council of Canada (2017), and the CanMEDS roles of the Royal College of Physicians and Surgeons of Canada (2017).

The first phase was to develop an assessment system for the Surgery Clerkship rotation at Queen’s University, based on rubrics with explicit behavioural descriptor. These tools would:

a. support faculty and residents to formulate judgments about a student’s clinical performance;
b. enhance the quality of feedback to students;
c. monitor students’ progress over the course of their surgery rotations;
d. identify students in need of additional support earlier in their clinical training.

Our second phase involved evaluating the acceptability, and the feasibility of the assessment tools, and to gather feedback from stakeholders to inform revisions to the tool. This phase included the creation of a new ambulatory clinic encounter card, based on faculty feedback. The third phase (not yet completed), will focus on developing a validity argument for use of these tools.

We met with faculty and residents to introduce them to the new assessment tool prior to its implementation. We used this opportunity to gather their feedback on tool content, ease of use, and purpose. Three round table discussions were held with 45 faculty and 45 residents in General Surgery, Urology, and Orthopedic Surgery. This collaboration between the development team and faculty members and residents resulted in the development of an additional assessment tool, the ambulatory clinic encounter card. Following implementation of both tools, student feedback was collected through a series of focus groups involving 60 participants.

## The Tools

**Figure 1.**
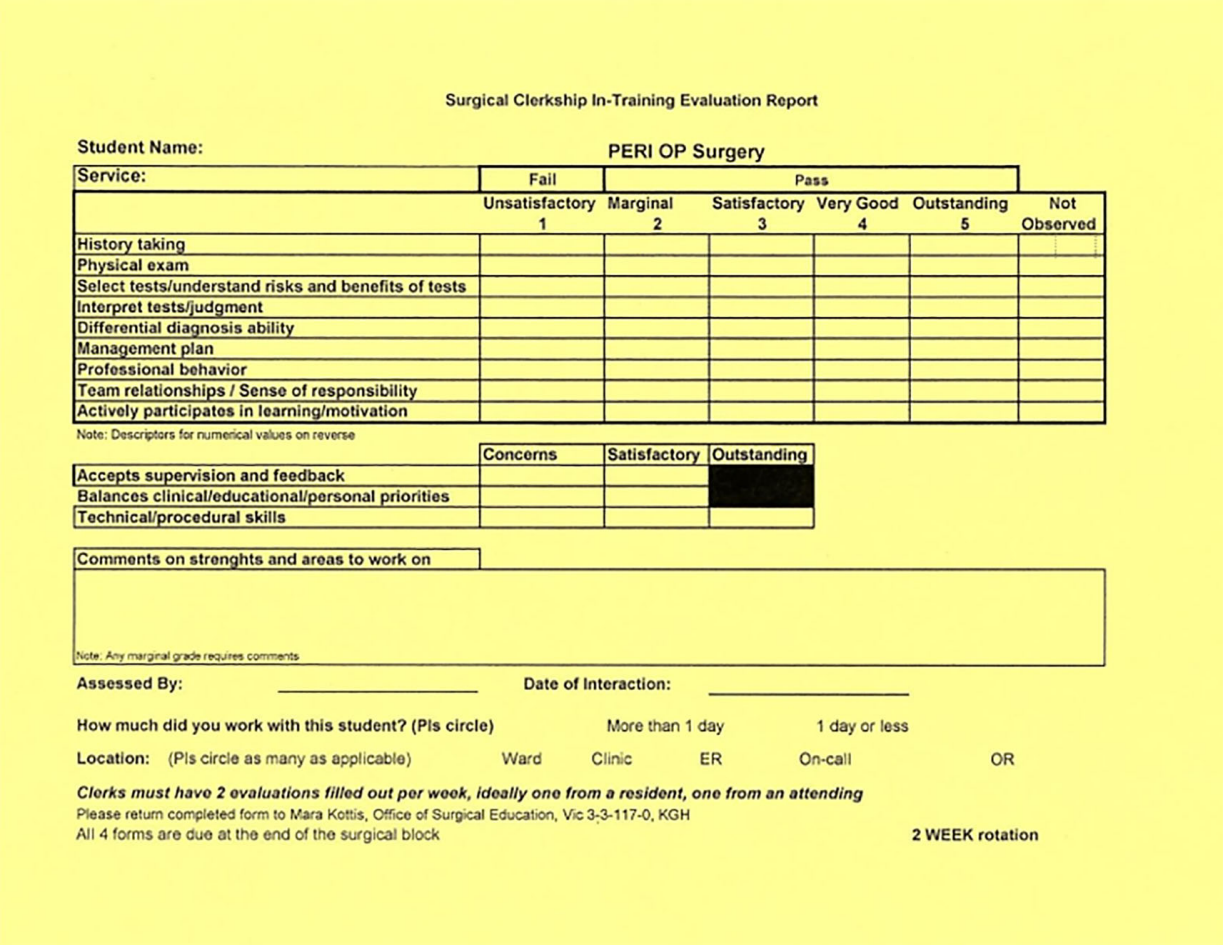
Previous Assessment Form

Our initial goal was to replace the existing workplace-based assessment form with two behavioural-anchored rubrics to increase specificity and quality of feedback. Previously, assessment for the Surgery Clerkship rotation consisted primarily of one tool which included both a Likert-scale and check box items ( Figure 1). Procedural skills were included as one check-box at the bottom of the form. In the pilot phase, however, we identified a need for a quick, scale-based assessment tool for half-day ambulatory clinic experiences. This led to the creation of the “Clinic Encounter Card.” As part of the overall development of the UGME procedural skills curriculum, a separate system of assessment for Clerkship procedural skills was developed concurrently (Patterson, Katsoulas, Hastings, Sanfilippo, & Jaeger, 2017). Ultimately, we created two rotation specific rubrics for the Surgery Clerkship.

**Figure 2.**
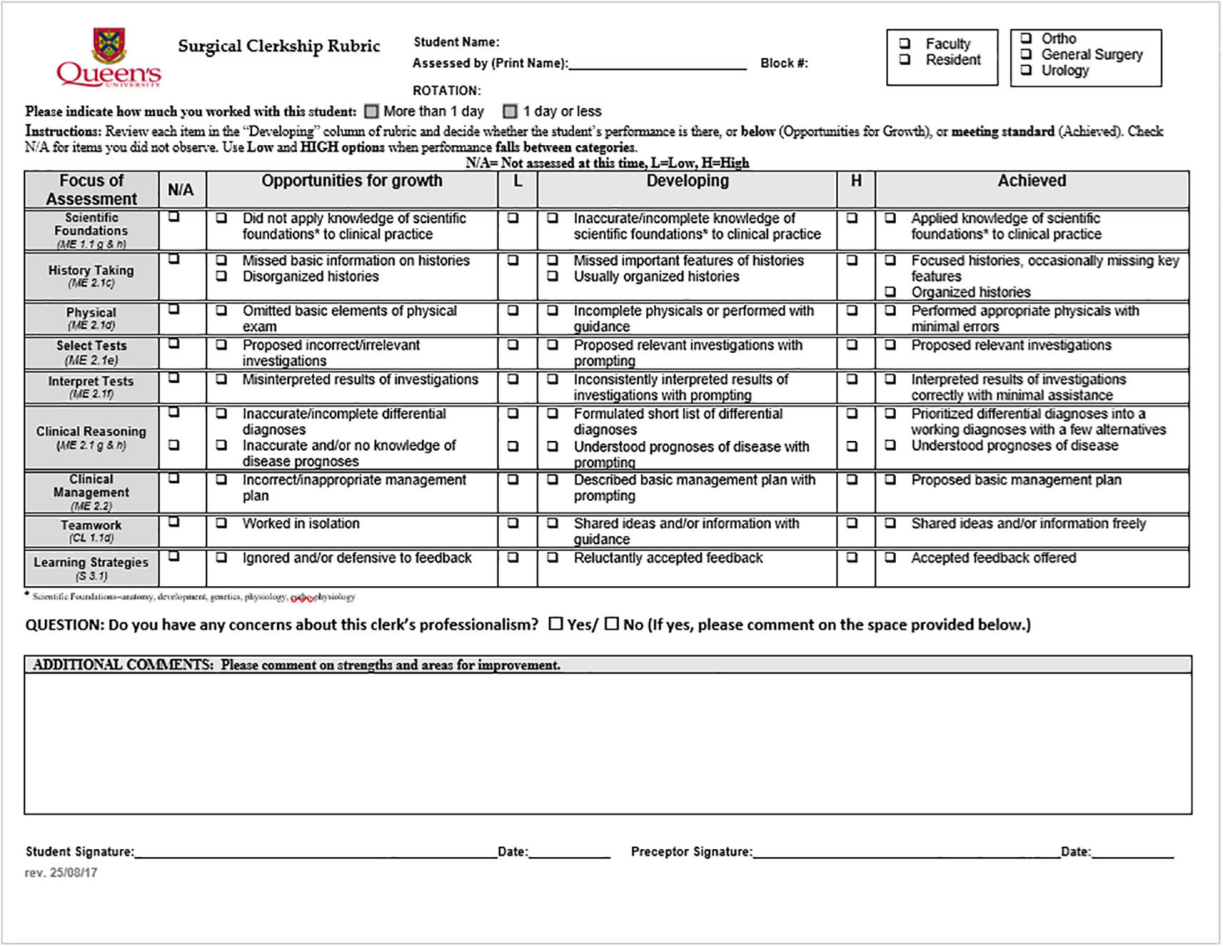
Surgical Clerkship Rubric

**Figure 3.**
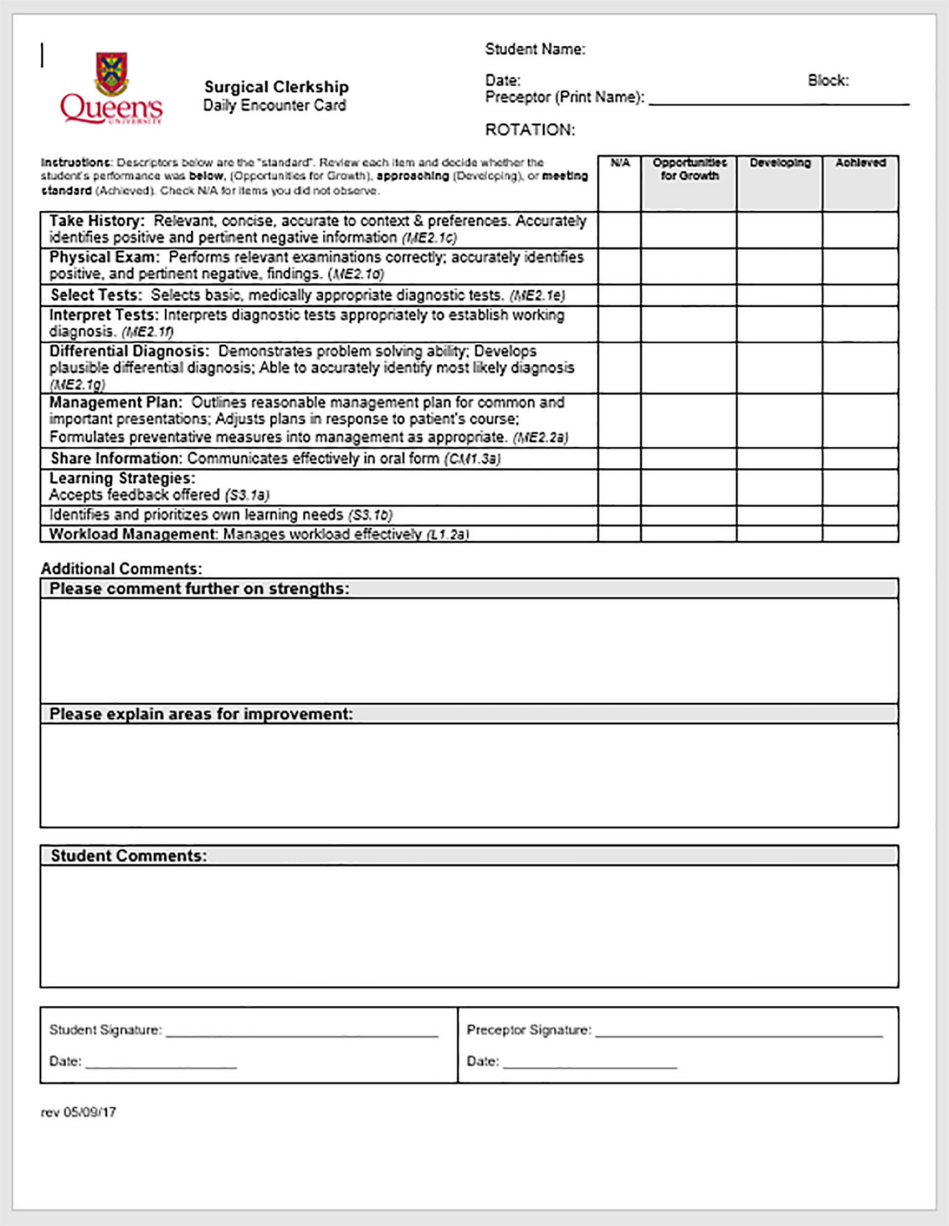
Surgical Daily Encounter Card

Under our new system, students must submit one Surgical Clerkship rubric ( Figure 2) for each week of rotation, for a total of six per block, and one Clinic Encounter Card per week ( Figure 3) for a total of six per block. The rubrics identify areas that provide “opportunities for growth” for students, areas they are still developing (i.e. “approaching standard”), and areas in which they are “achieving the standard. “The standard” here is defined by the behavioural anchors. The rubrics currently have high and low checkboxes to indicate what end of the spectrum within a level a student may be closest to. Faculty initially found this feature of the tool confusing. However, we have noted that these qualifiers are being used more frequently now, and we are continuing to collect data to make an informed decision on whether to keep these additional checkboxes. The “opportunities for growth” level identifies areas where learners may be struggling, and allows for early identification and focused interventions. For students who exceed the expected standard of performance, recognition is provided through the narrative comments included on these weekly assessments.

We chose to remove the option for “exceeds expectation/standard” because the purpose of the rubric is to identify students who are meeting the expected standards in any given area. We felt that the inclusion of ‘excellence’ set unrealistic expectations for both students and preceptors in terms of what students should be able to accomplish at this level of their clinical training. According to assessors’ feedback, the initial anchors for the rubric were targeted too high for the standards of a student in clerkship. One assessor mentioned that “even residents would not be able to accomplish some of these [behaviours]” in the “above standard” category, while another told us that “no medical student I have seen in 10 years meets your definitions of ‘above standard.’” Based on this feedback, we modified the language to better capture expectations for learners.

The ambulatory clinic Daily Encounter card was created based on assessor feedback from our Teaching, Learning and Innovations Director, who suggested that the level of detail in the Surgical Clerkship rotation rubric was difficult for faculty to assess when only interacting with students for a half-day in clinic. The encounter card focuses on behaviours that are more likely to be observed in the ambulatory clinical setting. This allows faculty to provide feedback to students even if they have only spent a limited period of time with the student. Encounter cards already in use in the Pediatric clerkship rotation were used as a model and tailored to fit the Surgical clinical learning environment. While students are only required to submit one clinic encounter card per week, they will often take the initiative to submit more than one. As medical students are typically focused on high achievement, some continue to struggle with the fact that there is no ‘outstanding’ or ‘excellent’ category of performance on the rubrics. Faculty use the narrative section of the rubrics to indicate the areas in which students excel, and are encouraged to put learners’ names forward for awards.

Taken together, these tools inform the mid-rotation and final-rotation assessments for students. Midway through their Surgical clerkship, and again at the completion students meet with the course director to discuss their progress. The formative information provided by the rubrics allows the course director to provide concrete feedback during these meeting, and to clearly articulate to students areas where they have met the standard for performance, and areas which require additional attention. These meetings are also an opportunity to communicate to students the areas in which they have excelled.

## Student Feedback

Feedback from students indicated that they find the rubrics a useful form of assessment; however, the usefulness of the tools depends on the resident or preceptor’s ability to provide quality actionable feedback, and varies with the time and effort that a resident or preceptor puts into completing the rubric, including the provision of narrative feedback. The following are selected student comments from the focus groups:

This [rubric] actually provides a formal end or discussion to the clinic which I don’t think I would have if we didn’t have the set number of rubrics to go on. . . It actually makes that feedback conversation happen where I think it wouldn’t necessarily happen otherwise.

[Rubrics] are fast.. structured and visual. It is good way of assessing performance.

Rubrics are primer for the [preceptor] because then they know what to tell you about.. If it was non-rubric [assessment form] I feel like the feedback would be more vague and more like, oh you are doing well for a clerk your level, and that would be it, versus this breaks it down a bit.

Preceptors would actually sit down and go through it, and it would be actually more like a meeting, so you would get feedback in that process.

Students also liked the new rubric for its concrete descriptions of levels of performance. They noted that the interpretation of the levels of previous tools could be variable depending on who completed it. One student suggested that previously “what [was] expected from you was so different based on who your residents were and who your preceptor was and what they wanted you to do. I like this [rubric] where you know what you are supposed to be doing.” The students particularly liked the behavioural-anchored descriptions for each level, noting that

It gives the people marking a bit more of an idea what a 1 [level] versus a 5 [level] means..Because what is good to one person might be different to another whereas with this [rubrics], they can think did they [clerks] describe management options with prompting, or [did] they [clerks] do it on their own kind of thing. So this is a bit more specific.. more thoughtful. The only concern with these forms is that they are very lengthy.

This concern was echoed by others who point out that the rubrics “are so dense that it takes a long time for preceptors to get through them,” and who worry that some preceptors may “get to the end of the form and neglect the comment section because they have spent so much time on the form.”

Students had the same reaction as faculty to the use of the initial clinical rotation rubrics in single-day clinic experiences. As one clerk noted, “for clinics [the rubric] became impractical sometimes.. Even though it is a good format.” While students recognized the value of the rubrics, they also recognized that they were not suitable for every situation. One student noted that “these [rubric] forms were not relevant for a quick half day clinic. The forms were too overwhelming for [faculty].” The introduction of the ambulatory clinic Daily Encounter Card, therefore, was very well-received. “These [daily encounter cards] were more user friendly,” one student explained. Another student observed that “the clinic encounter card . . . was a really good assessment tool, especially because we are usually in clinics for a half day with different preceptors. And so it is a good snapshot.” For another student, the encounter cards provided a balance between accuracy and practicality as he or she noted that “people are willing to do them because they are easy to read and it is an accurate assessment of what we are doing.”

## Next Steps

The new assessment tools have been in use in their current form for one year. We continue to receive faculty, resident, and student feedback, and we anticipate making ongoing revisions as part of an iterative process. For the past seven months we have been in the process. of evaluating the validity of these new assessment tools, and We hope to determine whether these new Surgical Clerkship assessment tools support faculty and residents in assessing students’ clinical performances, and whether the rubrics capture changes in patterns of performance over time for an individual student. Additionally, we plan to measure whether the use of these tools enhance the quality of feedback provided to students and whether assessments done using the rubrics correlate with other measures of student performance throughout the Clinical Clerkship component of the UGME curriculum.

## Take Home Messages

• Rubric-based assessment tools can be a valuable form of assessment within an EPA/CBME curricula.
• Assessor feedback during development of rubrics can assist in ensuring that metrics of performance accurately reflect reasonable expectations of students.
• Elimination of the “exceeds expectations” performance category requires that high achieving students be recognized in other ways, rather than assuming that they need to meet this level of performance in every domain.

## Notes On Contributors

Dr. Mila Kolar is an Assistant Professor and the Director of Undergraduate Surgical Education at Queen’s University.

Eleni Katsoulas is an Assessment and Evaluation Consultant with Undergraduate Medical Education at Queen’s University.

Dr. Ayca Toprak is a fifth-year resident in General Surgery at Queen’s University. She has expertise in Educational Assessment in Surgery.

Theresa Nowlan-Suart is an Educational Developer with Undergraduate Medical Education at Queen’s University

Dr. Lindsay Davidson is an Associate Professor and Director of Teaching, Learning and Innovation at Queen’s University

Dr. Andrea Winthrop is a Professor and Clerkship Director at Queen’s University

Dr. Amber Hastings-Truelove is an Education Researcher and consultant in the Faculty of Health Sciences at Queen’s University.

Dr. Denise Stockley is a Professor and Scholar in Higher Education at Queen’s University.
