# Supplementary material for: The Development and Implementation of New Assessment Tools for the Surgical Clerkship Rotation
Source: MedEdPublish (2016). 2018 Jan 4;7:4. [Version 1] doi: 10.15694/mep.2018.000004.1 (PMC10711974; doi:10.15694/mep.2018.000004.1)
Supplement: Peer review history

# Reviewer report: Reviewer response for version 1

**DOI:** 10.21956/mep.19477.r28749

**Published:** 2018-01-06

**Authors:** Tripti Srivastava

**Affiliations:** 1 Datta Meghe Institute of Medical Sciences Deemed University

This review has been migrated. The reviewer awarded 4 stars out of 5 One more significant step towards competency - based curriculum. The rubric worked out for surgical clerkship is logically explained, particularly ‘High’ and ‘Low’ is a well thought option. Though the rubric seems exhaustive, a sensitization about the same to assessors will probably suffice to make them comfortable with its practice. The ‘Surgical Daily encounter card’ is another rubric suggested within the article particularly for a half day clinic. This rubric collates few competencies together for grading. Though the competencies are inter-related, it may be more helpful to rate each competency separately that will provide a better picture of the learners level of achievement. Much eagerly waiting for the observations of the third phase. Recommended read.

Reviewer Expertise:

NA

No decision status is available

# Reviewer report: Reviewer response for version 1

**DOI:** 10.21956/mep.19477.r28748

**Published:** 2018-01-04

**Authors:** Trevor Gibbs

**Affiliations:** 1 AMEE

This review has been migrated. The reviewer awarded 4 stars out of 5 I enjoyed reading this paper and thought that it covered a very important area for development: I look towards further research from this group on how this project develops.I feel that the principles that this paper are based upon are very clear and very appropriate and attempt to form an important link with the performance, its assessment and improvement of the students as they progress towards graduation. It will be interesting to read about how the faculty ( preceptors etc.) really find this form of assessment with a greater depth of discussion, how it interfaces with the on-going issue between time spent in service and teaching, whether all the outcomes expected can be achieved and assessed within the time allocated to surgery and of course the predicted validity of the method.

Reviewer Expertise:

NA

No decision status is available
